# Percutaneous Fixation of Anterior Column Acetabular Fracture in a Renal Transplant Recipient

**DOI:** 10.1155/2013/842390

**Published:** 2013-06-17

**Authors:** Halil Ceylan, Ozgur Selek, Ahmet Y. Sarlak

**Affiliations:** ^1^Department of Orthopaedics and Traumatology, Van İpekyolu State Hospital, 65100 Van, Turkey; ^2^Department of Orthopaedics and Traumatology, Kocaeli University School of Medicine, Umuttepe, 41380 Kocaeli, Turkey

## Abstract

Renal transplantation, performed per million population, ranges from 30 to 60 in developed countries. The transplanted kidney is generally placed in iliac fossa; therefore the treatment procedure of the pelvic trauma in these patients should be selected carefully. The gold standard technique for the treatment of displaced acetabulum fractures is open reduction and internal fixation. Our patient had received a living-related-donor renal transplant due to chronic renal failure. In the second year of transplantation, she had been injured in a motor-vehicle accident, and radiographs showed a right acetabular anterior column fracture and left pubic rami fractures. The patient was treated with percutaneous fixation techniques and at one year of postoperative period there was no evidence of degenerative signs and the clinical outcome was good. Beside having the advantage of avoiding dissection through the iliac fossa by the standard ilioinguinal approach, percutaneous techniques, with shorter surgical time, decreasing soft tissue disruption, and the potential for early discharge from hospital might be ideal for a renal transplant recipient carrying a higher risk of infection. Percutaneous fixation of selected acetabular fractures in a renal transplant recipient would presumably have the potential to decrease the morbidity associated with traditional open surgical procedures.

## 1. Introduction

Acetabular fractures are rare with an incidence of 3 patients/100000 year [1]. Anterior column fractures were reported to be the least frequent type which accounts of 12.3*℅* of all acetabular fractures [2]. The incidence of renal transplantation performed per million population, ranges from 30 to 60 in developed countries [3]. It is obvious that the coincidence of an anterior column acetabular fracture in a patient with renal transplant is extremely rare. As the transplanted kidney is generally placed in iliac fossa, the treatment procedure of the pelvic trauma in these patients should be selected carefully [4].

The gold standard technique for the treatment of displaced acetabulum fractures is open reduction and internal fixation as described by Judet et al. [5]. In an attempt to overcome the morbidity of extensile surgical approaches, percutaneous fixation of the pelvis has been receiving increasing attention [6]. In pelvic trauma patients percutaneous techniques have been specially recommended in patients with polytrauma [7, 8], severe open injuries, extensive closed degloving injuries [9, 10], and in elderly with medical comorbid condition [6, 11]. Percutaneous screw fixation of acetabulum fractures is a relatively new procedure, and the indication for its use is not fully defined [12].

The purpose of this case report in to suggest a new indication for percutaneous fixation of acetabulum fractures in a renal transplant recipient.

## 2. Case Report

A 31-year-old woman had received a living-related-donor renal transplant in 2010 due to chronic renal failure secondary to chronic granulomatous tubulointerstitial nephritis. She had an uneventful postoperative course and had been receiving tacrolimus 1 mg/day, mycophenolic acid 360 mg/day, and prednisolone 10 mg/day as maintaining treatment. Her allograft function was normal with a serum creatinine level of 1.24 mg/dL.

**Figure 1:**
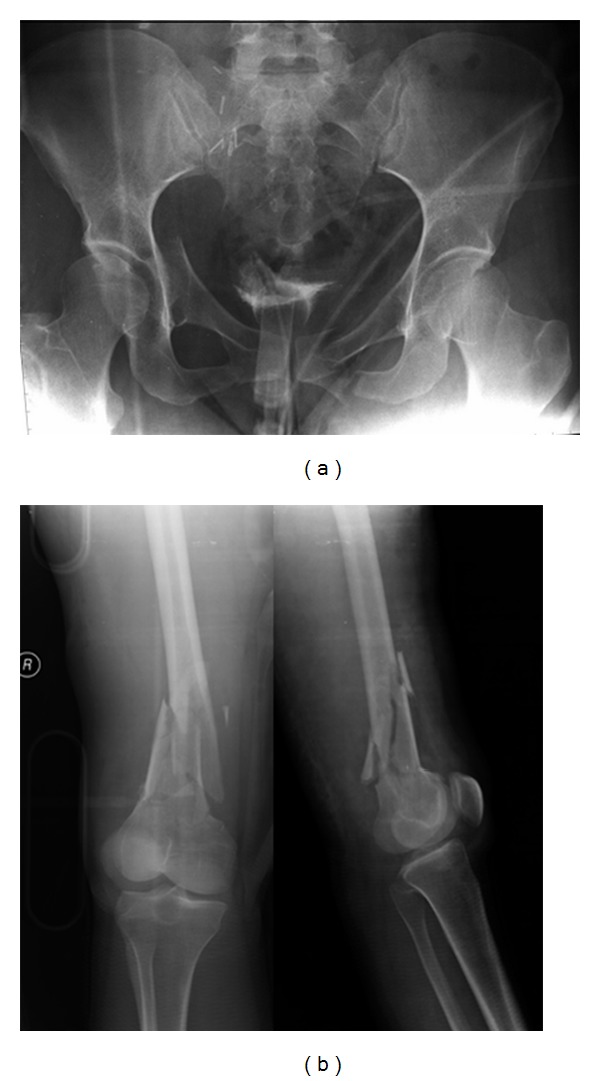
X-ray in the emergency room showing (a) the right-sided anterior column and the left-sided pubic rami fractures of acetabulum. (b) The right-sided distal femoral metaphyseal complex (OTA 33-D3) fracture.

**Figure 2:**
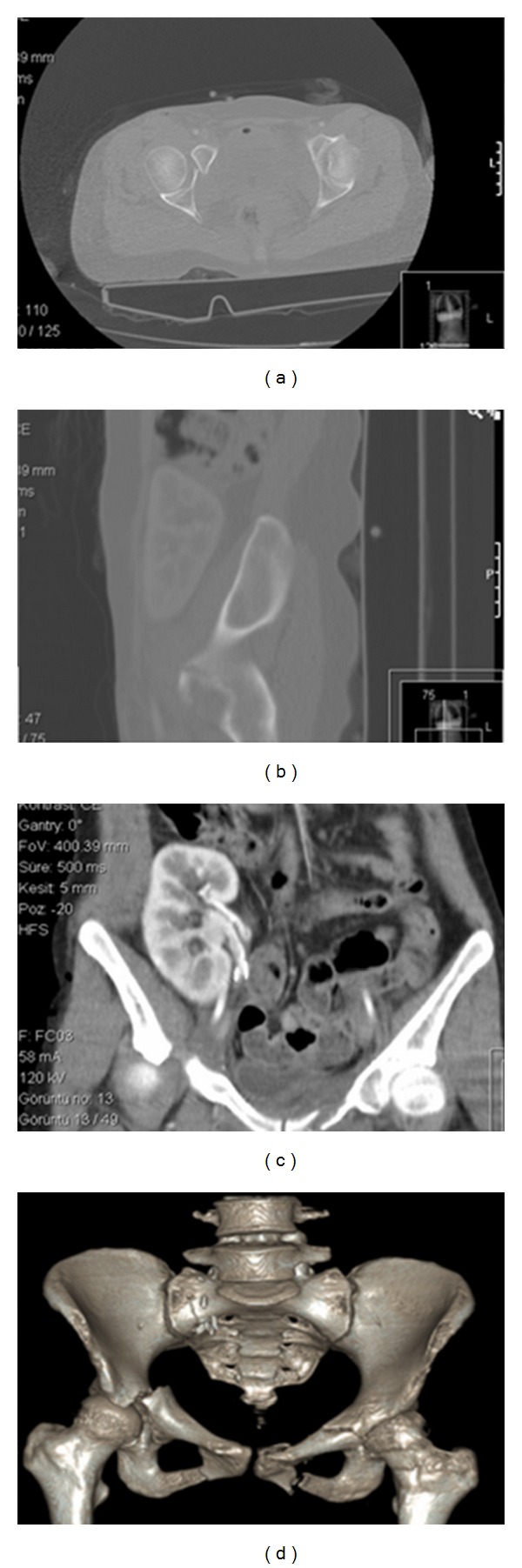
(a, b) Computed tomography (CT) showing the fracture of acetabulum. (c) Transplanted kidney is seen at coronal section of CT. (d) 3D CT view of fracture.

In the second year of transplantation, she had been injured in a motor-vehicle accident and was taken to the emergency room in our hospital. On admission the systolic blood pressure was 140/82 mm Hg; pulse was 96 beats/min; respiratory rate was 24 breaths/min; and oxygen saturation was normal at room air. Subsequent examinations were also stable. She presented with a Glasgow Coma Scale score of 15 without obvious head or neck trauma. She complained of severe pain in right hip and right thigh. Physical examination revealed tenderness around the right hip and the left pubis as well as obvious deformities of the patient's right thigh and also Morel-Lavelle lesion at the anterolateral aspect of the right thigh and at the trochanteric region. There was no neurovascular deficit. Systemic examination including genitourinary tract was normal. Initial laboratory examination revealed hemoglobin level 11.7 gm/dL, serum potassium 3.58 mmol/L, and serum creatinine 1.24 mg/dL. Abdominal ultrasound was negative for the presence of free fluid. Radiographs showed a right acetabular anterior column fracture, left pubic rami fractures, and also right distal femur metaphyseal complex (OTA 33-D3) fracture (Figure 1). Computed tomography demonstrated a 7 mm displacement of the anterior column fracture (Figure 2).

At the fourth day of the trauma, the patient was placed in a supine position on a radiolucent table in the operating room. Under general anesthesia at first closed reduction and retrograde intramedullary nailing were done for distal femur fracture. And then closed reduction maneuver was tried by using traction and internal-external rotation for acetabulum fracture. The standard C-arm fluoroscopy of Judet iliac and obturator oblique views was used in order to confirm reduction. As the fluoroscopic views showed inadequate reduction, the reduction was achieved by a clamp through a small incision as described by Starr et al. [13].

**Figure 3:**
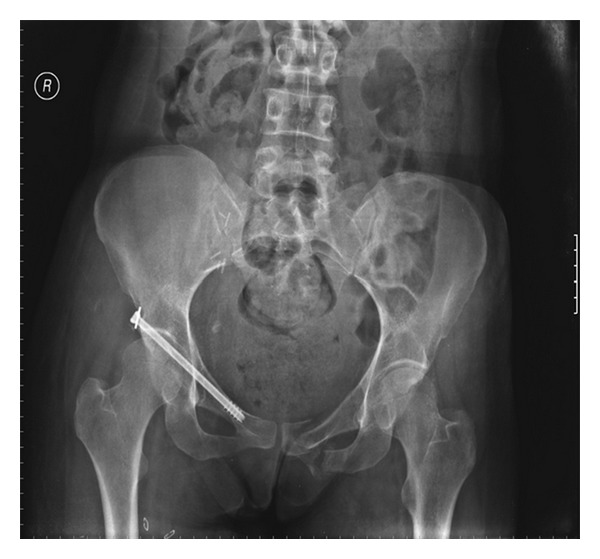
Postoperative X-ray: percutaneous fixation of anterior column fracture via 7,3 cannulated screw.

After obtaining anatomical reduction, a soft tissue sleeve was used to insert a guide wire passing from supra-acetabular area to superior pubic rami. Multiple fluoroscopic views were obtained in order to confirm the place of inserted guide wire. A 7,3 mm cannulated screw was then placed over the guide wire (Figure 3).

**Figure 4:**
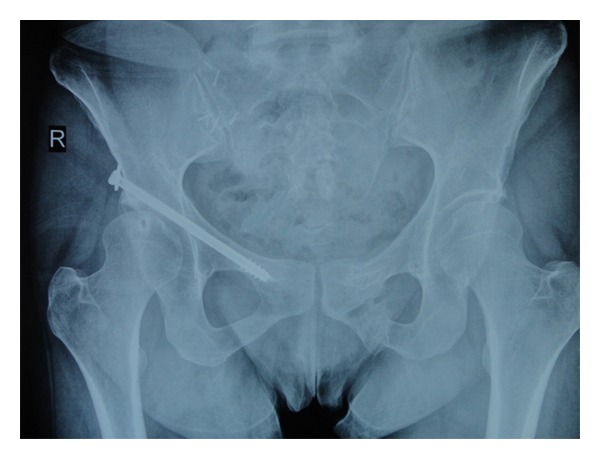
X-ray after one year of operation showing the good clinical outcome.

Postoperatively, subcutaneous low molecular weight heparin, compression stocking, and pneumatic compression devices were used for ten days prophylactically. Physical therapy was begun at the first postoperative day, and the patient was instructed to bear weight “foot-flat” with a walker. The patient was instructed for full weight bearing two months postoperatively. The radiograph at one year of postoperative period showed no evidence of degenerative signs, and the clinical outcome was good (Figure 4). 

The closed internal degloving injury was treated by three separate debridements after operative fixation. The wound was treated with split-thickness skin grafts.

## 3. Discussion

Renal transplantation is the best choice of treatment in end stage renal failure in order to prolong survival and improve the quality of life. As the survival rate for renal transplant approaches 86*℅* at 10 years, transplant recipients are expected to return to more active lifestyles, including a significant risk for becoming a trauma victim [14].

The first step should be to rule out injury to the transplanted kidney in anterior pelvis. There may be direct injuries to the renal parenchyma or to the urinary bladder. Contrast enhanced CT cystograms and renal duplex examination were reported to be indicated to identify renal, urethral and urinary bladder injury and to assess renal blood flow and function [15]. Immunosuppressive treatment should be balanced according to the condition of the patient; especially in the event of renal trauma [15].

In a renal transplant recipient the widely recognized complications include susceptibility to infection, poor wound healing, capillary fragility, osteoporosis, hypertension, aseptic osteonecrosis, and glucose intolerance [16]. As well as having the advantage of avoiding dissection through the iliac fossa, percutaneous techniques, with shorter surgical time, decreasing soft tissue disruption, and the potential of early discharge from hospital might be ideal for a renal transplant recipient carrying a higher risk of infection [8, 13, 17, 18].

Percutaneous fixation under image guidance with cannulated screws was recommended in cases of minimally displaced transtectal acetabular fractures, high anterior column fractures, and posterior hemitransverse fractures of the anterior column [6]. While anterior column fractures were reported to the ideal of minimally invasive surgery, as the major fracture component in perpendicular to the supraacetabular axis, there have been only three prevision studies that investigated percutaneous fixation of anterior column fractures exclusively [17, 19, 20]. In general high anterior column fractures with the anterior superior iliac spine attached to the externally rotated proximal fragment were more easily managed in an antegrade fashion, reduced with a rigid pin to the displaced fragment acting as a joystick [17]. In low anterior column fractures retrograde percutaneous screws might be preferred to more easily control the mobile superior pubic fragment, that is, to be fixed to the stable proximal fragment.

Due to low fracture pattern of our case and also the 7 mm preoperative fracture displacement which was accepted to be unfeasible for closed percutaneous technique, the management might be more easy in a retrograde fashion [18]. We think that in a patient with renal transplant it may be mandatory to choose the antegrade semiopen or closed technique to avoid injury by an inadvertent, pin screw passage directed laterally from pubic tubercle in the retrograde technique. Ideal insertion side might be more easily controlled by a safe distance from the tip of the renal transplant in the antegrade percutaneous screw fixation technique.

Percutaneous fixation of selected acetabular fractures in a renal transplant recipient would presumably have the potential to decrease the morbidity associated with traditional open surgical procedures.
